# Comparative Genomics of Wild Bee and Flower Isolated *Lactobacillus* Reveals Potential Adaptation to the Bee Host

**DOI:** 10.1093/gbe/evz136

**Published:** 2019-07-01

**Authors:** Hoang Q Vuong, Quinn S McFrederick

**Affiliations:** 1 Department of Entomology, University California Riverside; 2 Department of Plant Pathology and Microbiology, University California Riverside

**Keywords:** *Lactobacillus*, symbiosis, bees, host–microbe, population genomics, comparative genomics

## Abstract

Symbiosis with bacteria is common across insects, resulting in adaptive host phenotypes. The recently described bacterial symbionts *Lactobacillus micheneri*, *Lactobacillus timberlakei*, and *Lactobacillus quenuiae* are found in wild bee pollen provisions, bee guts, and flowers but have small genomes in comparison to other lactobacilli. We sequenced, assembled, and analyzed 27 new *L. micheneri* clade genomes to identify their possible ecological functions in flower and bee hosts. We determined possible key functions for the *L. micheneri* clade by identifying genes under positive selection, balancing selection, genes gained or lost, and population structure. A host adherence factor shows signatures of positive selection, whereas other orthologous copies are variable within the *L. micheneri* clade. The host adherence factors serve as strong evidence that these lactobacilli are adapted to animal hosts as their targets are found in the digestive tract of insects. Next, the *L. micheneri* clade is adapted toward a nutrient-rich environment, corroborating observations of where *L. micheneri* is most abundant. Additionally, genes involved in osmotolerance, pH tolerance, temperature resistance, detoxification, and oxidative stress response show signatures of selection that allow these bacteria to thrive in pollen and nectar masses in bee nests and in the bee gut. Altogether, these findings not only suggest that the *L. micheneri* clade is primarily adapted to the wild bee gut but also exhibit genomic features that would be beneficial to survival in flowers.

## Introduction

Bees are important for wild and agricultural ecosystems but are also in decline (Potts et al. 2010; Burkle et al. 2013). Research on the bee microbiome has increased in the past decade, due to the new interest in bee health (Engel et al. 2016). Eusocial corbiculate bees, a monophyletic clade of bees in family Apidae which notably includes bumble bees and honey bees, have a specific core microbiome that is important for bee health (Martinson et al. 2011; Li et al. 2015; Kwong et al. 2017). The core microbes confer pathogen defense by inducing host immune functions and directly antagonizing pathogenic microorganisms (Vojvodic et al. 2013; Engel et al. 2016). Strains of *Gilliamella apicola* have the capability to metabolize carbohydrates toxic to the host (Zheng et al. 2016). Honey bee and bumble bee core gut lactobacilli ferment a wide variety of simple carbohydrates in the gut, whereas *Snodgrassella* can convert fermented products to pyruvate for metabolism (Kwong, Engel, et al. 2014; Kwong, Mancenido, et al. 2014).

Bumble bees and honey bees serve as powerful models for microbiome studies (Kwong et al. 2017), but findings of bumble bees and honey bees do not apply to all species of bees. Noneusocial apids (family: Apidae) and noncorbiculate bees (families: Andrenidae, Colletidae, Halictidae, Megachilidae, Melittidae, and Stenotritidae) (all hereafter referred to as wild bees) harbor microbiomes with fewer taxa, are less dense with bacteria, and have less consistent communities compared with honey bees and bumble bees (Martinson et al. 2011; McFrederick et al. 2014). For example, the dominant taxon that associates with megachilid bees (Megachilidae) and halictid bees (Halictidae) is the heterofermentative *Lactobacillus micheneri* (McFrederick et al. 2017). Initially observed as a single species, *L. micheneri* has since been described as three distinct species: *L. micheneri*, *Lactobacillusquenuiae*, and *Lactobacillustimberlakei* that we refer to collectively as the *L. micheneri* clade (McFrederick et al. 2018). To avoid confusion, the *L. micheneri* species will be referred as “*L. micheneri*.”

Despite having reduced genomes compared with other lactobacilli, *L. micheneri* clade bacteria associate with multiple flower and wild bee species (McFrederick et al. 2017, 2018). There is no pattern in host-specificity, as evidenced by the detection of *L. micheneri* and *L. timberlakei* in both megachilid and halictid bees, which is a likely result of horizontal transmission (McFrederick et al. 2017). The ecological function and genomic capabilities of the *L. micheneri* clade are, however, unknown. *Lactobacillus kunkeei* is sister to the *L. micheneri* clade, but *L. kunkeei* is absent or at most present in scarce densities in wild bees (McFrederick et al. 2017; Rothman et al. 2018). *Lactobacillus kunkeei* is typically only detected from flowers and the crops and colony surfaces of honey bees and is not considered part of the honey bee core microbiome (Anderson et al. 2013; Kwong et al. 2017). The lactobacilli that are considered part of the core microbiome in honey bees consists of the homofermentative *Lactobacillusapis* and *Lactobacillusmellis* and their relatives, which are distantly related to *L. micheneri* and *L. kunkeei* (McFrederick et al. 2013). Wild bees are predominantly associated with heterofermentative lactobacilli, whereas honey bees are transiently colonized by heterofermentative lactobacilli but colonized at high density with homofermentative lactobacilli. The closest relatives to *L. micheneri* and *L. kunkeei* are lactobacilli that have been isolated from flowers (Kawasaki et al. 2011), fermented vegetable drinks (Chiou et al. 2018), and sourdough starter (Vogel et al. 2011), but are suggested to be associated with insects (Duar et al. 2017). Because *L. micheneri* inhabits a novel niche, it has likely evolved new functions that facilitate its association with flowers and wild bees. We compared 27 new *L. micheneri* clade genomes with publicly available *Lactobacillus* genomes to detect signatures of adaption which led to the association with wild bees. We identified genes under positive selection, balancing selection, genes gained or lost, and population structure. These genes are candidates of important functions involved in colonizing wild bees and surviving in flowers.

## Materials and Methods

### Sample Collection

We used DNA from cultures originally isolated in McFrederick et al. (2017). We isolated seven samples from *Megachile rotundata* pollen provisions collected in Wellsville, UT, by plating a phosphate buffered saline serial dilution of pollen provisions on de Man Rogosa Sharpe (MRS, De Man et al. 1960) plates fortified with 2% or 20% fructose. After plating the pollen provisions, we purified single isolates by subculturing individual colonies three successive times. We extracted DNA from the pure cultures using the Qiagen Blood and Tissue kit protocol with lysozyme.

### Genome Sequencing, Annotation, Assembly, and Reference Genome Sequence Access

To prepare sequencing libraries, we used the Illumina Tru-Seq DNA PCR-free kit, following the manufacturer’s instructions. For libraries with low DNA concentrations, we used Illumina’s universal primers to amplify libraries in a PCR programed for 98 °C for 3 min and 10 cycles of 98 °C for 10 s, 60 °C for 30 s, and 72 °C for 30 s with a final extension at 72 °C for 5 min. Once normalized, we sequenced the genome libraries with the Illumina MiSeq with 2x300 V3 reagents. After sequencing, we used the A5 pipeline (ver: 05222015) to assemble reads into contigs and scaffolds (Coil et al. 2015). Once assembled, we annotated our genomes using RAST (Aziz et al. 2008). We used CheckM on the Kbase browser-based tool to assess the completeness and contamination of the draft genomes (Parks et al. 2015; Arkin et al. 2018; McFrederick et al. 2018). After annotation, we aligned all *L. micheneri* clade genomes from McFrederick et al. (2018) and this study using SPINE and ClustAGE to identify accessory and core genome elements of the clade and each species with multiple isolates sequenced (Ozer et al. 2014; Ozer 2018). We uploaded our RAST annotations to the KEGG automatic annotation server (Kanehisa and Goto 2000; Moriya et al. 2007; Minoru Kanehisa et al. 2016). We accessed relative *Lactobacillus* genomes (*N* = 22) with the Pathosystems Resource Integration Center (PATRIC) database (Wattam et al. 2017). We used the Shapiro–Wilk test and Mann–Whitney–Wilcoxon test for pairwise comparisons of nonnormally distributed sample sets for GC, genome size and coding DNA sequence (CDS) in the R base software package (R Core Team 2018). Genomic feature graphs were created using the R package ggpubr (Kassambara 2017).

### Ortholog Detection and Verification

We used OrthoMCL to detect orthologs in the 52 genomes using a recommended inflation value of 1.5 (Li et al. 2003). We used our 30 *L.micheneri* clade genomes along with 22 related lactobacilli. Using *Lactobacillus plantarum* as an outgroup, we reconstructed the phylogenetic history of the focal lactobacilli using the concatenated supermatrix of single copy orthologs (*N* = 583 orthologs) with RAxML (Stamatakis 2014) and the GTRGAMMA model for the entire supermatrix. We verified all genes by modeling protein structure with SWISS-model (Waterhouse et al. 2018) and BlastP searches against the UniProt database (UniProt Consortium 2017).

### Ancestral Genome State Reconstruction

We analyzed the ortholog and singleton gene frequency table of the 52 genomes to reconstruct the ancestral genome state using the program Count with the Wagner Parsimony assumption (Gene Gain Penalty = 2) (Csurös 2010). In addition to reconstructing the ancestral genome state of each clade of the core genome phylogeny (supplementary fig. 1*A* and *B*, Supplementary Material online), Count determined the genes that were gained or lost between each clade and branch tip. Using a custom Bash parser, we crossed-referenced genes gained or lost between each clade and branch tip with the annotation.

### Detecting Positive Selection in the *L. micheneri* Clade Using the Branch-Site Model

We searched ortholog sequences for nonsynonymous mutations which serve as signatures of positive selection using the PAML package codeML (Yang 2007). We searched for orthologs with a significantly better fit to models that include positive selection compared with the null, neutral selection. For each ortholog, we compared the likelihood ratio values of the two models to determine whether the resulting comparison yielded a significant *P* value. If the selection model significantly fit positive selection for the ortholog of interest, we identified amino acid sites under positive selection using the Bayesian empirical Bayes inference (Zhang et al. 2005). We used a phylogeny containing all *Lactobacillus* genomes in this study to search the ortholog sequences for sites under positive selection in the *L. micheneri clade* (supplementary fig. 1*A* and *B*, Supplementary Material online). Next, we searched for ortholog sequences with sites under positive selection between *L. micheneri* and *L. timberlakei* and the rest of the *L. micheneri* clade with *L. quenuiae* as the outgroup.

### Allele Frequency and Population Structure Analyses

We retrieved all orthologs from *L. micheneri* and *L. timberlakei*, for which we have multiple isolates. We analyzed the allele frequency of orthologs of the *L. micheneri* clade species with DNAsp (Librado and Rozas 2009). We created a single nucleotide polymorphism (SNP) map in the VCF format using Bowtie2, Samtools, Bcftools, and Vcftools to analyze the population structure across the *L. micheneri* Hlig3 genome as a reference (Li et al. 2009; Danecek et al. 2011; Langmead and Salzberg 2012). Next, we inputted the SNP map into the R package “PopGenome” and used the sliding window method (1-kb windows and steps) to calculate *F*_st_ (Pfeifer et al. 2014). We excluded the genome of the type strain of *L. quenuiae* as we have isolated only one strain of *L. quenuiae* so far, which would not allow us to make accurate inferences in population structure. We calculated *F*_st_ for each contig containing variant calls between *L. micheneri* and *L. timberlakei* genomes. Based on prior usage and empirical determination (Zhang et al. 2015; Hardigan et al. 2017), we selected windows that have an *F*_st_ above the 95th percentile or 1,000-bp windows and steps with an *F*_st_ of at least 0.932 between *L. micheneri* and *L. timberlakei*.

## Results

### Draft Genome Quality and Genome Features of *L. micheneri* Clade Bacteria

**Fig. 1.**
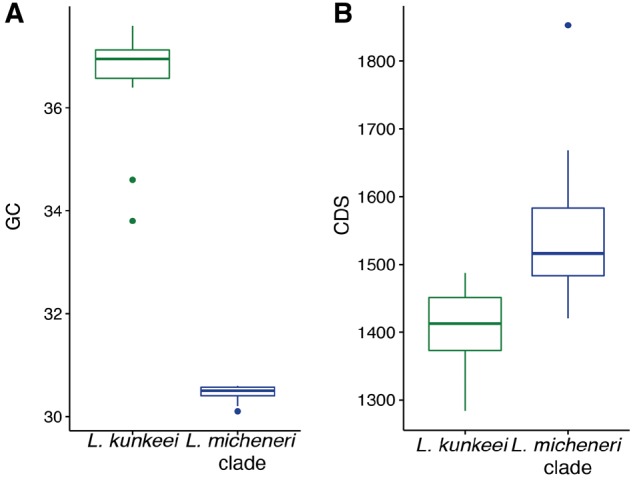
—*Lactobacillus micheneri* clade bacteria significantly differ from *L. kunkeei* in genomic GC% (*A*) and CDS (*B*). Means and *P* values are in table 1.

Based on the CheckM results, we find that all the draft genomes have a completeness of 95% or greater. All but three genomes had no reported contamination, only HV_6, HV_10, and HV_23 had low contamination of 0.062%, 0.053%, and 1.5%, respectively. We tested all genomic feature data for normality using the Shapiro–Wilk test. The Shapiro–Wilk test indicated that all but two data sets were nonnormal distributions (table 1)*. Lactobacillus micheneri* clade genomes (*N* = 30) were significantly more AT-biased (*P* < 0.001, Mann–Whitney–Wilcoxon test, fig. 1*A* and table 1) than the genomes of sister taxa *L. kunkeei*. *Lactobacillus micheneri* had a significantly larger number of CDSs per Mb than *L. kunkeei* genomes, because there was significant difference in the number of CDS (*P* < 0.001, Mann–Whitney–Wilcoxon test; fig. 1*B* and table 1*A*) and genome size (*P* < 0.05, Mann–Whitney–Wilcoxon test; fig. 1*B* and table 1*A*) between the *L. micheneri* clade and *L. kunkeei*. *Lactobacillus timberlakei* (*N* = 9) had significantly larger genomes and significantly higher counts of CDS than *L. micheneri* (Genomes: *P* < 0.001; CDS: *P* < 0.001, Mann–Whitney–Wilcoxon test; fig. 1*C* and *D* and table 1). Notable accessory genome features from the CLUSTAGE analysis in the *L. micheneri* clade include a polysaccharide biosynthesis gene cluster only in *L. timberlakei* (supplementary table 2, Supplementary Material online; accessory genome element 15 and 51). Other features include one chitinase that is annotated as a fibronectin/fibrinogen-binding protein in only *L. micheneri* HV_63, HV_64, HV_67, and *L. quenuiae* type HV_6 (supplementary table 2, Supplementary Material online; accessory genome element 99.1, 25.10), and a pectate lyase copy present only in *L. micheneri* and *L. quenuiae* (supplementary table 2, Supplementary Material online; accessory genome element 97.7).

**Table 1:** Statistical Analyses of Genomic Features: GC% (*A*), CDS (*B*), and Genome Size (*C*) between *L. kunkeei* and the *L. micheneri* Clade; *L. micheneri* and *L. timberlakei*

Compared Species/Groups	Comparison Mean	Shapiro–Wilk *P* Value	Mann–Whitney *W*	*P* Value
(*A*) GC%				
*L. kunkeei* (*N* = 15) and *L. micheneri* clade (*N* = 30)	36.8%, 30.5%	0.0002771, 0.0004398	480	2.238e-08
*L. micheneri* species (*N* = 20) and *L. timberlakei* (*N* = 9)	30.5%, 30.4%	0.0001767, 0.008338	135	0.02629
(*B*) CDS				
*L. kunkeei* and *L. micheneri* clade	1,407, 1,540	0.4868, 0.0007096	29.5	1.276e-06
*L. micheneri* species and *L. timberlakei*	1,502, 1,619	0.04671, 0.006994	11	3.834e-05
(*C*) Genome size				
*L. kunkeei* and *L. micheneri* clade	1,536,381, 1,500,683	0.1275, 7.966e-05	341	0.02045
*L. micheneri* species and *L. timberlakei*	1,468,371, 1,567,777	0.0005775, 0.02314	18.5	8.158e-04

### Ortholog Detection and Core Ortholog Phylogeny of Distant and Close Relatives of *L. micheneri*

OrthoMCL detected 969 single copy orthologs shared across the 30 genomes from the *L. micheneri* clade. In addition, 1,118 and 1,092 orthologs were shared within the *L. micheneri* and *L. timberlakei* species, respectively. When comparing the *L. micheneri* clade with close relatives such as *L. kunkeei* and distant relatives such as the *L. plantarum* clade we detected 583 orthologs. We aligned and concatenated the amino acid sequence of these 583 orthologs to create a supermatrix for input to RAxML to construct an ortholog phylogeny of 11 *Lactobacillus* species (supplementary fig. 1*A* and *B*, Supplementary Material online). Of the 583 orthologs, 32 orthologs did not pass alignment for the branch-site analysis, leading to only 551 orthologs analyzed. The resulting phylogeny showed 100% bootstrap support of the three species as monophyletic groups (supplementary fig. 1*A* and *B*, Supplementary Material online).

### Gene Gain and Loss Reconstruction

**Fig. 2.**
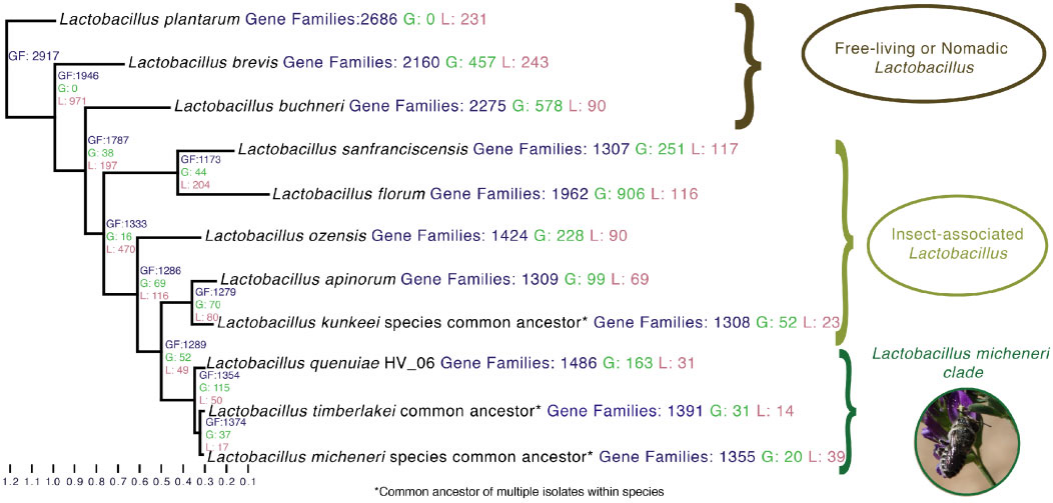
—Genome reduction from the common ancestor of *Lactobacillus sanfranciscensis* and *Lactobacillus micheneri* is conserved in the present-day species. Gene gain and loss was calculated with the Wagner Parsimony assumption in the Count program (Gene Gain Penalty = 2). Numbers that are labeled GF (blue) are the total counts of gene families present in the genome or ancestral state. Numbers labeled G (green) and L (red) are the counts of gene families gained and lost in the genome or ancestral state, respectively.

We used the phylogeny created from the ortholog analyses as input to reconstruct gene gain and loss (supplementary fig. 1*A* and *B*, Supplementary Material online). The gain or loss of genes may represent an important adaptation to the bee or flower host environments for *L. micheneri* via a loss or gain of possible function*.* The common ancestor of the *L. micheneri* clade gained 115 genes and lost 50 genes. The 115 genes gained included pectate lyase, which has been biochemically characterized as a bioactive extracellular enzyme in *Lactobacillus* (Sakellaris et al. 1989). Other examples include two copies of beta-hexosaminidase identified as a dispersin B (dspB) (Donelli et al. 2007), a biphenyl degradation gene (Ishigooka et al. 1986), and a cobalt–zinc–cadmium resistance gene involved in heavy metal transport (Zhai et al. 2017) (fig. 2 and supplementary table 3, Supplementary Material online). Compared with the common ancestor to the *L. micheneri* clade, *L.quenuiae* gained 158 genes, including a gene annotated by UniProt and SWISS-model as a fibronectin/fibrinogen-binding protein, and lost 31 genes (fig. 2 and supplementary table 3, Supplementary Material online). The common ancestor of *L. timberlakei* gained 31 genes, including the YoeB/YefM toxin anti-toxin system and genes involved in the persistence of cells in the presence of antibiotics and the absence of nutrients (Wang and Wood 2011) (fig. 2 and supplementary table 3, Supplementary Material online).

### Metabolism and Biosynthetic Pathway Reconstruction of the *L. micheneri* Clade

Using the KEGG annotations, we analyzed the biosynthetic and metabolic pathways of the type strains of *L. micheneri*, *L. timberlakei*, and *L. quenuiae* described in McFrederick et al. (2018) and outgroups. We found several consistent patterns across the *L. micheneri* clade. 1) There was little to no hexose metabolism outside of glucose, sucrose, and fructose as maltose and sucrose phosphorylases are absent (table 2 and supplementary fig. 2*A*, Supplementary Material online). 2) Although the pathway to produce tyrosine or phenylalanine is incomplete, the *L. timberlakei* type strain can convert 3-dehydroquinate to 4-hydroxyphenyl pyruvate, an intermediate that is a potential lactic acid fermentation substrate (supplementary fig. 2*B*, Supplementary Material online). However, the other two *L. micheneri* clade type strains can only convert chorismate to prephenate (supplementary fig. 2*B*, Supplementary Material online). 3) None of the type strains has the full pathway to synthesize thiamine from purine or biosynthesize biotin (supplementary fig. 2*C* and *D*, Supplementary Material online).

**Table 2:** Carbohydrate Metabolism Absent (White) or Present (Blue) Summary Based on KEGG and RAST Annotations of *Lactobacillus* Type Strains in Study

	Amylose, and Mannitol	Xylose	Arabinose, Lactose, Cellobiose, and Galactose	Mannose	Trehalose	Dextrin	Maltose	Sucrose, Glucose, and Fructose	Sorbitol	Beta-Hexosanimidase	Pectate Lyase
*L. plantarum*											^a^
*L. buchneri*					^b^	^b^			^b^		
*L. brevis*					^b^	^b^			^b^		
*L. sanfranciscensis*						^b^			^b^		
*L. florum*									^b^		
*L. ozensis*									^b^		
*L. apinorum*									^b^		
*L. kunkeei*							^a^		^b^		
*L. quenuiae*									^b^		
*L. timberlakei*									^b^		
*L. micheneri*									^b^		

a Found in other genomes of species in study.

b Not by KEGG but by RAST annotation.

### Population Structure Analyses of *L. micheneri* and *L. timberlakei*

**Fig. 3.**
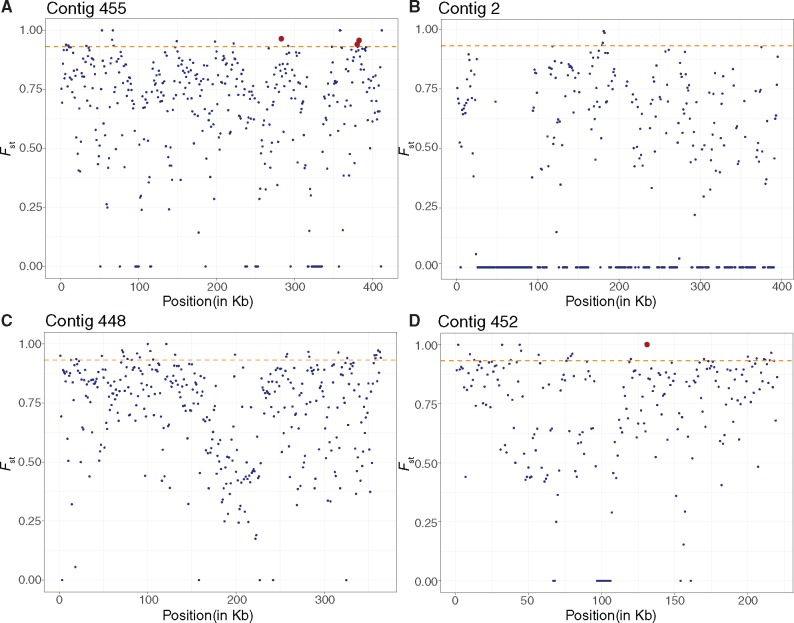
—Population structure analyses in 1-kb windows and 1-kb steps between *Lactobacillus micheneri* and *Lactobacillus timberlakei* mapped to four *Lactobacillus micheneri* Hlig3 contigs reveal strong population structure. The orange dashed line represents the threshold for the 1-kb windows with the 95th percentile *F*_st_ (0.932). Red dots represent gene regions discussed in text, listed from left to right as: LtaS, cshA, MurR, and kup. Regions where *F*_st_ above the 95th percentile is listed in supplementary table 4, Supplementary Material online.

During our population structure analysis, we removed four *L. timberlakei* genomes (HV_04, HV_09, HV_12, and HV_27) from the overall data set. Although these genomes were assembled with a small number of contigs (15–29) and acceptable coverage (15.31–31.36), they had the fewest number of error-corrected reads (112,820–272,407) and percentage of nucleotides (44.29–63.82%) passing quality filtering via the A5 pipeline. Because our population structure analyses use raw reads instead of assemblies from error-corrected reads, the uncorrected raw reads could lead to a missing variant call at a variant site. Including these genomes could, therefore, lead to inaccurate *F*_st_ readings. With the remaining genomes, we calculated *F*_st_ of *L. timberlakei* and *L. micheneri* to detect diverging genes between two species. *F*_st_ is the fixation index between a subpopulation and the total population or two populations, at a single SNP or a nucleotide range or window. An *F*_st_ value of 1 means that the SNP is fixed in one population and not in another. An *F*_st_ value of 0 indicates no differentiation between the two populations. Genes with *F*_st_ values above the 95th percentile are of interest as their respective function may be under selective pressure resulting in divergence between *L. micheneri* and *L. timberlakei.* We detected eleven 1-kbp windows where *F*_st_ = 1, which include the coding sequence of seven protein-coding genes and one promoter site (fig. 3 and supplementary table 4, Supplementary Material online). The protein-coding genes where *F*_st_ = 1 include a kup K^+^ uptake system, a gene involved in osmoregulation, and pH tolerance (Trchounian and Kobayashi 1999). We retrieved 86 genes from the 70 windows that were in the 95th percentile of *F*_st_ values. These genes include a cold-shock DEAD-box protein A (cshA) a temperature sensitive regulator (Hunger et al. 2006), lipoteichoic acid synthase type IIb (ltaS), and UDP-*N*-acetylmuramoylalanyl-d-glutamyl-2,6-diaminopimelate–d-alanyl-d-alanine ligase (murF) (supplementary table 4, Supplementary Material online).

### *Lactobacillus m* *icheneri* Clade Species Ortholog Allele Frequency Analyses

We calculated Tajima’s *D* for each ortholog within *L. micheneri* and *L. timberlakei* (Librado and Rozas 2009). A significant positive Tajima’s *D* score suggests that the respective ortholog is undergoing balancing selection, where more than expected alleles are maintained in the population. Alternatively, genome-wide significant positive Tajima’s *D* scores suggest that the population has suddenly contracted or is splitting. A negative Tajima’s *D* score suggests that the population is either recovering from a bottleneck (genome-wide) or that the respective gene has recently undergone a selective sweep, where fewer than expected alleles are maintained. The 30 *L. timberlakei* orthologs with a significantly negative Tajima’s *D* score include glutamine transport ATP-binding protein (glnQ), a transporter involved in osmotolerance and pH resistance (Cotter and Hill 2003) and DEAD-box ATP-dependent RNA helicase (cshB), a temperature sensitive regulator (Hunger et al. 2006) (supplementary table 5, Supplementary Material online). The 125 orthologs with a significant positive Tajima’s *D* score in *L. timberlakei* include the glycine betaine ABC transport system (opuAC) involved in osmotolerance and pH resistance (Papadimitriou et al. 2016) (supplementary table 5, Supplementary Material online). The 473 orthologs of *L. micheneri* with a significant negative Tajima’s *D* score includes betaine transport protein (betT) involved in glycine transport for osmotolerance and pH resistance. There were orthologs with significant positive Tajima’s *D* scores in both *L. timberlakei* and *L. micheneri* (supplementary table 5, Supplementary Material online). These genes include the glycine betaine ABC transport system genes (opuAA\AB) involved in osmotolerance and pH resistance (Papadimitriou et al. 2016) (supplementary table 5, Supplementary Material online).

### Positive Selection Detection Using the Branch-Site Model

Across the *L. micheneri* clade, 108 genes out of 551 tested orthologs showed signatures of positive selection (supplementary table 6, Supplementary Material online). Fixation of nonsynonymous mutations is rare as amino acid sequences are conserved under purifying selection. However, amino acid sequence changes can result from selection pressure arising from adapting to novel or changing environments. Genes in the *L. micheneri* clade exhibiting signatures of positive selection may, therefore, be important in adaptation to the wild bee and/or flower niche. Genes undergoing positive selection in only *L. micheneri* or *L. timberlakei* may also be important for niche differentiation between these two species. The DEAD-box ATP-dependent RNA helicase (cshA), a gene involved in temperature sensitive gene regulation (Hunger et al. 2006), had the lowest *P* value from the positive selection analysis (supplementary table 6, Supplementary Material online). The other 107 genes included 2,3-butanediol dehydrogenase, a protein involved in producing a volatile important for resisting gut acidification in honey bee core lactobacilli (Lee et al. 2015) and fibronectin/fibrinogen-binding protein (supplementary table 6, Supplementary Material online). When searching for orthologs containing sites under positive selection in the genomes of *L. micheneri* and *L. timberlakei* we found 8 and 12 orthologs, respectively (supplementary table 6, Supplementary Material online). In *L. micheneri*, the eight genes include the cadmium, zinc, and mercury transporting ATPase (copB) (Solioz et al. 2010) (supplementary table 6, Supplementary Material online). In *L. timberlakei*, the genes included heavy metal transporter manganese transport protein mntH (Makui et al. 2000) and a heme-dependent catalase that is orthologous to a heme-dependent catalase involved in reactive oxidative stress in *L. plantarum* (Knauf et al. 1992) (supplementary table 6, Supplementary Material online).

## Discussion

The genomes of the *L. micheneri* clade show signatures of selection and novel functions that underlie the ability of these bacteria to thrive in nutrient-rich environments. Given the small size of their genomes, the ability of these bacteria to persist on flowers and proliferate in pollen and nectar masses inside bee nests and inside the bee gut is remarkable. Although all these environments are rich in plant metabolites, the bee gut immune system exerts further pressure. It is therefore perhaps not surprising that their genomes reveal adaptations to harsh, nutrient-rich environments as well as animal host-specific adaptations (supplementary table 7, Supplementary Material online). Compared with the honey bee-associated lactobacilli, the *L. micheneri* genomes are more reminiscent of pathogens or endosymbionts with more compact and more AT-biased genomes with less carbohydrate metabolism capabilities (fig. 1 and table 1) (Ellegaard et al. 2015; Zheng et al. 2016).

Although ours is the first comparative genomics study of wild bee-associated lactobacilli, previous work on honey bee core microbes and *L. kunkeei* allows interesting contrasts to our results. For example, Tamarit et al. (2015) found that *L. kunkeei* has short generation times. Ellegard et al. (2015) found that the honey bee gut lactobacilli genomes are rich in carbohydrate metabolism and transport genes. *Gilliamella* can utilize a variety of monosaccharides that are toxic to honey bees (Zheng et al. 2016) and *Gilliamella* and *Snodgrasella* may work together to provide nutrients to their host (Kwong 2014).

Honey bees and wild bees share common ancestry but differ in their life histories. Although honey bees are active year-round, wild bees often have short periods of adult activity and spend unsuitable seasons in a quiescent state (Michener 2007). The phenology of wild bees and flowers may therefore cause a bottleneck which reduces gene flow occurring in the *L. micheneri* clade. Although not an adaptation, this may be why the *L. micheneri* clade genomes are more AT-rich than *L. kunkeei* and core honey bee gut lactobacilli (Ellegaard et al. 2015). In addition to AT-biases, the *L. micheneri* clade have more compact genomes than core honey bee gut lactobacilli (Ellegaard et al. 2015). Additionally, compared with within-colony, social transmission in honey bees (Kwong and Moran 2016) horizontal transmission occurring at flowers may be more important for maintenance of *L. micheneri* in wild bee communities which include both solitary and primitively eusocial host taxa (McFrederick et al. 2017).

These similarities and differences in host biology may explain why we find both convergence and novelty in symbiont functions by honey bee microbes and the *L. micheneri* clade. *Gilliamella* sp. has pectate lyase genes which allow them to digest pollen intines and increase honey bee worker weight (Zheng et al. 2017). The presence of pectate lyase in the *L. micheneri* clade may mean that these bacteria are important for wild bee nutrition, as nearly all female bees digest pollen to build protein reserves necessary for egg production (Cane 2016; Cane et al. 2017). *Gilliamella* sp., however, has more enzymes for digesting nectar and pollen saccharides compared with the *L. micheneri* clade (Engel et al. 2012; Zheng et al. 2016) and the *L. micheneri* clade is depauperate in regards to carbohydrate metabolism genes.

Inhibition of pathogenic organisms observed in *L. kunkeei* (Vásquez and Olofsson 2009; Forsgren et al. 2010) may be possible in the *L. micheneri* clade. dspB may be used to inhibit unwanted biofilm-forming bacteria or pathogens in synthetic environments (Donelli et al. 2007) and *L. micheneri* clade bacteria may use dspB to regulate the dispersal of their biofilms in response to environmental stressors such as reactive oxygen species (ROS) produced by competitors or hosts (Stacy et al. 2014). Fungi may be inhibited by the *L. micheneri* clade by d- and l-lactic acid production and can be enhanced by hydroxyphenyllactate and phenyllactate in *L. timberlakei*. These compounds are all inhibitory to fungi and inhibition has been documented in *Lactobacillussanfranciscensis* (Lavermicocca et al. 2000) and *L. kunkeei* (Vojvodic et al. 2012). The *L. micheneri* clade has gained, lost, and refined functions that are likely behind its ability to thrive in the new wild bee guts and pollen provisions.

### Host Binding and Adherence Factors

In silico prediction and annotation of the genes involved in host binding and adherence factors provides evidence supporting the hypothesis that the *L. micheneri* clade is adapted to the bee gut. The *L. micheneri* clade and their outgroups have genes predicted to be fibronectin/fibrinogen-binding proteins that target animal specific proteins, suggesting that they are more likely to have a role in animal association (Henderson et al. 2011). Specifically, in insects, fibrinogen is known to be present in the peritrophic matrix of the midgut (Hu et al. 2012), whereas both fibrinogen and fibronectin transcripts are expressed in the hind gut (Neira Oviedo et al. 2008). The fibronectin binding gene of the *L. micheneri* clade is under positive selection, potentially improving *L. micheneri* clade interaction with bees compared with the outgroups. Copies of fibronectin/fibrinogen-binding protein are also variable in the *L. micheneri* clade, which may improve their ability to establish in certain strains of animal hosts (Buck et al. 2005). The production of polysaccharides or exopolysaccharides in *L. timberlakei* may allow more efficient host immune evasion and adhesion in bees guts compared with *L. quenuiae* or *L. micheneri* (Leigh and Coplin 1992; D’Haeze and Holsters 2004; Lee et al. 2016).

### Metabolic Capabilities Correlate to Nectar and Pollen-Rich Niches

Our data suggest that the *L. micheneri* clade thrive best in pollen and nectar-rich environments such as wild bee pollen provisions or wild bee guts. We previously found *L. micheneri* to be abundant in these environments (McFrederick et al. 2017). However, our findings do not exclude the possibility that these adaptations are important to their survival in flowers, where these lactobacilli are also found (McFrederick et al. 2017). The *L. micheneri* clade appears to be streamlined to utilize nectar carbohydrates, as they can only digest sucrose, glucose, and fructose, the three most predominant sugars of nectar (Nicolson 1998). The presence of pectate lyase genes is also remarkable, as the closest related lactobacilli to contain these genes is *L. plantarum* (Sakellaris et al. 1989).

### Adaptation to pH, Osmotolerance, and Temperature in Bee Guts and Flowers

Although *L. micheneri* and *L. timberlakei* are very closely related (McFrederick et al. 2018), we find divergence in genes involved in osmotolerance, pH, and temperature tolerance. Such genes are also involved in response to rapid osmolarity or pH changes. Divergence in these functions suggests that these bacteria have adapted to wild bee guts and pollen provisions. These adaptations can be especially important as different sections of the bee gut have different osmotic potentials and pH (Peng and Dobson 1997). In addition to different osmotic potentials or pHs in bee guts, there are variable osmolarities in different flower nectars (Nicolson 1998). These genes may, therefore, underlie the divergence between *L. micheneri* and *L. timberlakei.*

Temperature-sensitive transcriptional regulators under positive selection may be important as wild bees and flowers do not thermoregulate their nests as honey bees and bumble bees can, leading to temperatures dropping during nights or cold days (Graham and Patterson 1982; Liu et al. 2005). Differences in host biology also correlate with the optimum growth temperature of core honey bee lactobacilli, which is the optimum maintained temperature in honey bee hives (35 °C, Jones et al. 2004; Olofsson et al. 2014), as opposed to 32 °C for the *L. micheneri* clade (McFrederick et al. 2018). Further studies on these genes and their roles in niche differentiation in the *L. micheneri* clade are needed to test these hypotheses.

### Detoxification and Oxidative Stress Response

The *L. micheneri* clade appears to be adept at detoxification, as we found signatures of toxin tolerance or mitigation in their genomes. Toxins such as heavy metals and biphenyl can bioaccumulate in flowers and are therefore likely to occur in pollen provisions and bee guts in areas with contaminated soils (Hladun et al. 2015; McFrederick et al. 2017). Although catalase genes are extremely uncommon in lactobacilli, they may be important for the *L. micheneri* clade because both flowers and bees produce ROS that repel microbes (O’Brien et al. 2012; Buchon et al. 2014). Additionally, the signatures of positive selection in *L. timberlakei* in catalase suggest a competitive advantage when exposed to ROS compared with *L. micheneri* or *L. quenuiae*. Finally, *L. timberlakei* may be more competitive than *L. micheneri* or *L. quenuiae* under antibiotic or nutrient-limiting stress due to the additional toxin anti-toxin genes in *L. timberlakei* (Wang and Wood 2011).

## Conclusion

Although experimental verification is needed, the genomic data presented here suggest several possible mechanisms by which *L. micheneri* clade bacteria may benefit their bee hosts. Given the largely absent metabolic and biosynthetic capability of the *L. micheneri* clade, it is unlikely that *L. micheneri* clade wild bee associates are biosynthetic mutualists. This is not surprising as all the necessary nutrients are largely present in pollen and nectar (Nicolson 1998; Roulston and Cane 2000). None the less, based on genomic in silico predictions, the *L. micheneri* clade bacteria have the potential to aid pollen digestion, detoxification, thrive in acidic environments, inhibit potential pathogens, and establish in wild bees. However, as our findings are limited to only genomic data, it is possible that *L. micheneri* clade bacteria are either commensal or detrimental to their hosts. If commensal, *L. micheneri* may exploit the nutrient-rich bee host environment, and bees may act as vectors for *L. micheneri* to spread to more flowers and bees. Finally, if parasitic, *L. micheneri* may exploit the nutrients in nectar and pollen at the expense of the bee hosts. Regardless, these reduced genomes offer strong clues as to how these bacteria persist and thrive in harsh but nutrient-rich niches.

## Supplementary Material

Supplementary data are available at *Genome Biology and Evolution* online.

## Supplementary Material

evz136_Supplementary_Data

## Notes

**Data deposition:** This project has been deposited at NCBI under the accession PRJNA484967.
