# Supplementary material for: Comparative Genomics of Wild Bee and Flower Isolated Lactobacillus Reveals Potential Adaptation to the Bee Host
Source: Genome Biol Evol. 2019 Jul 1;11(8):2151–61. doi: 10.1093/gbe/evz136 (PMC6685495; doi:10.1093/gbe/evz136)
Supplement: evz136_Supplementary_Data [file evz136_supplementary_data.zip › Supplementary_Figures_1-2.docx]

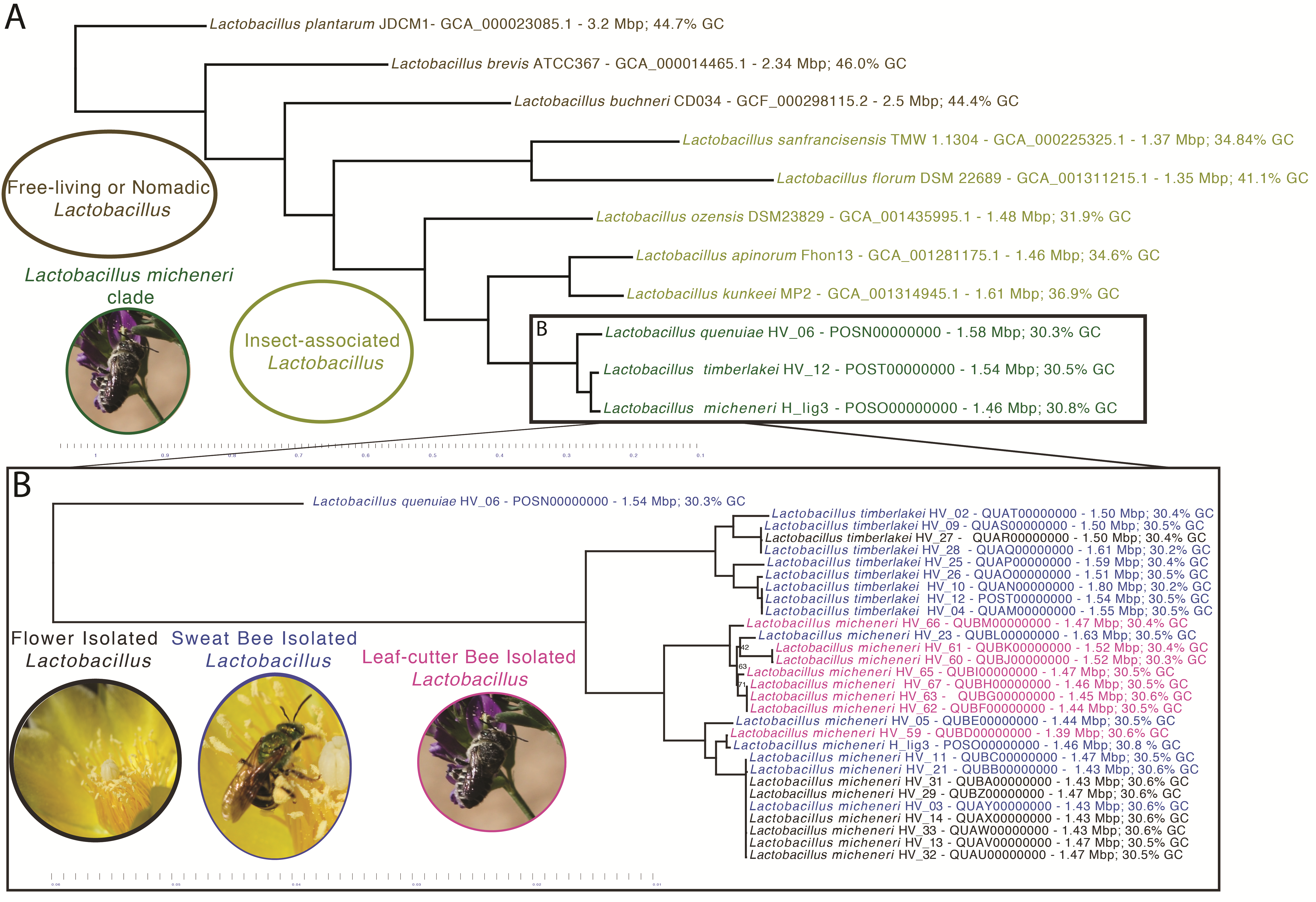


Supplementary Figure 1: The wild bee-associated *L. micheneri* clade is sister to *L. kunkeei*. (A) Phylogeny using an aligned super-matrix of 583 protein orthologs of *Lactobacillus* (A). Isolates color-coded by host isolation source in (A) and (B), as indicated by the color of circle and text in legend. (B) Maximum likelihood phylogeny based on an aligned super-matrix of 969 protein orthologs of *Lactobacillus micheneri* clade (B).

A)


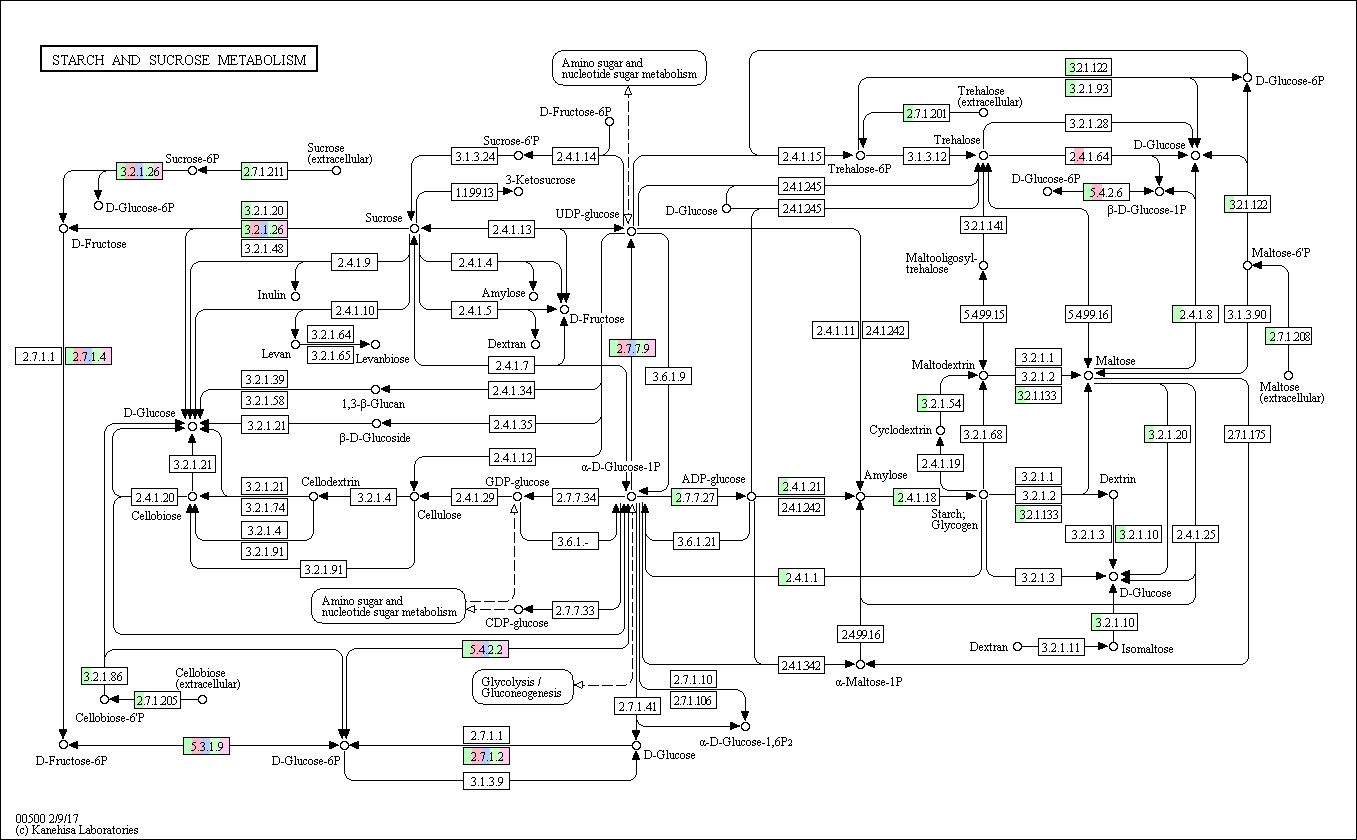


B)


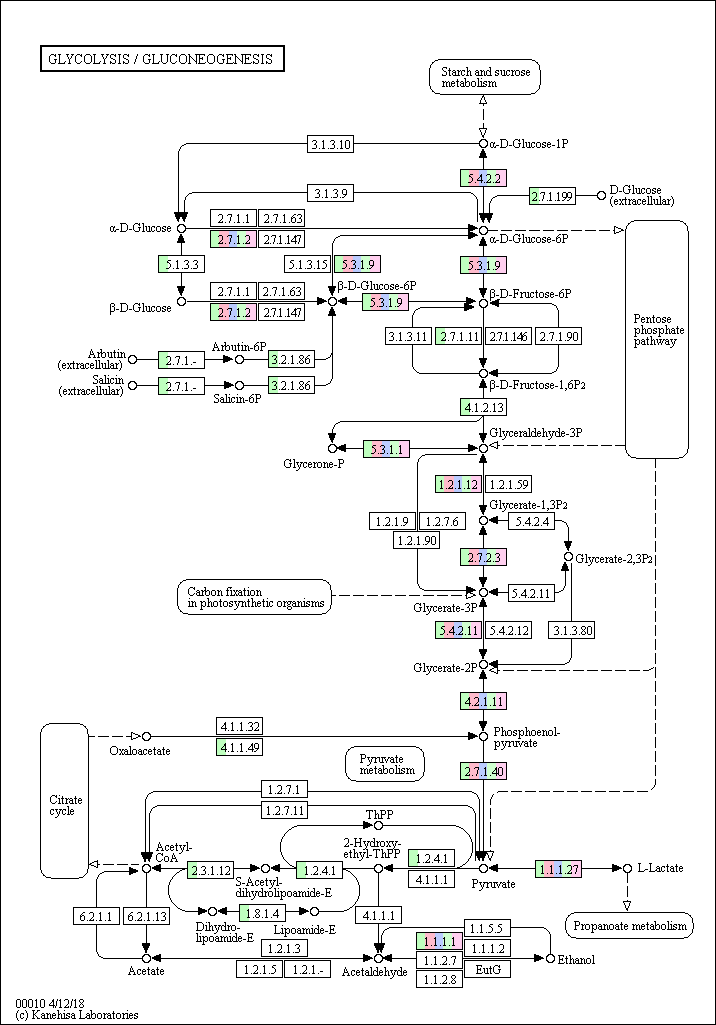


C)


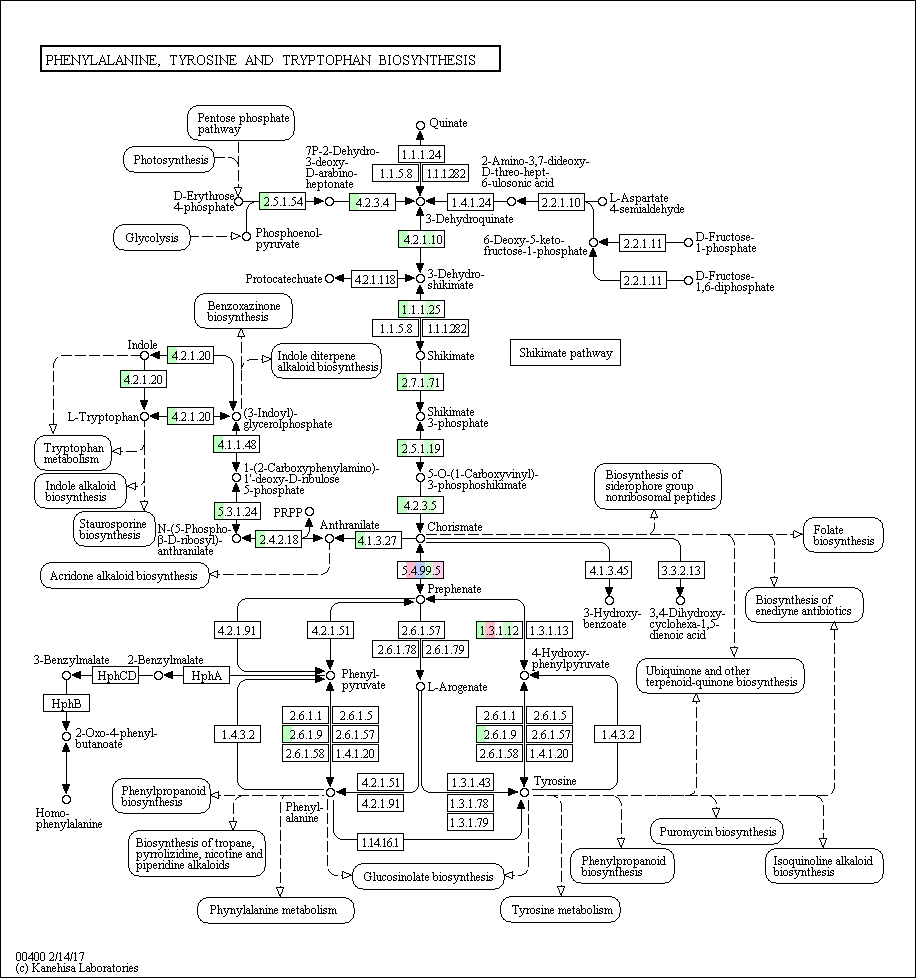


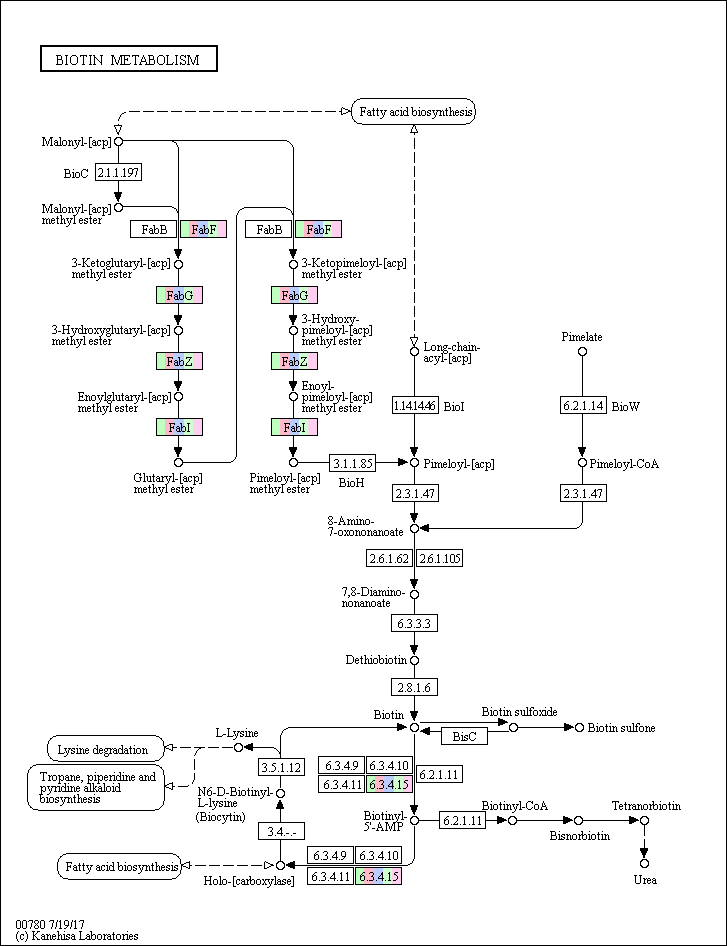
D)

E)


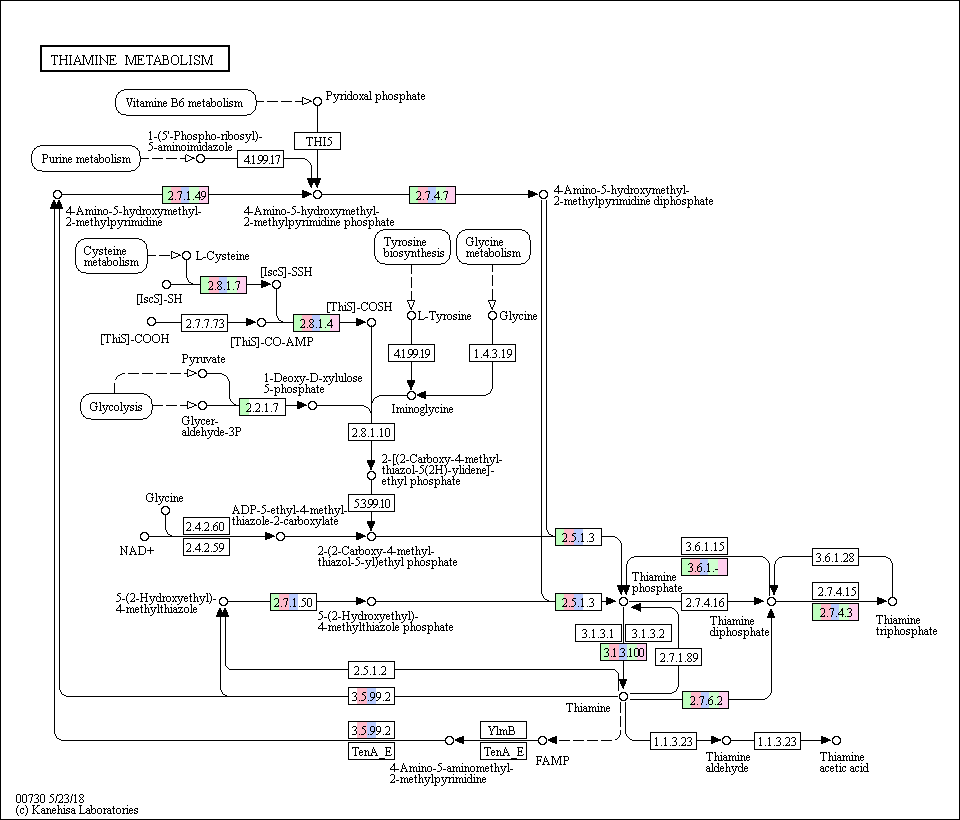


Supplementary Figure 3: A) Starch and Sucrose, B) Glycolysis, C) Aromatic Amino Acids Metabolism, D) Thiamine Metabolism, and E) Biotin metabolism Metabolism KEGG tables of *Lactobacillus* taxa. The presence of an enzyme involved in a pathway of its respective box is colored five colors, which corresponds to the five selected *Lactobacillus.*  *Lactobacillus plantarum* JDM01 is in dark green on the furthest left position, dark red for *L. kunkeei* MP2 on the second from the left position, blue for *L. quenuiae* HV_06 in the middle position, light green for *L. timberlakei* on the second from the right position, and light red for *L. micheneri* Hlig in the furthest right position. If the enzyme is absent in the *Lactobacillus* the corresponding position in a box representing an enzyme is colored white.
